# Locations and patterns of meiotic recombination in two-generation pedigrees

**DOI:** 10.1186/1471-2350-10-93

**Published:** 2009-09-17

**Authors:** Jason C Ting, Elisha DO Roberson, Duane G Currier, Jonathan Pevsner

**Affiliations:** 1 Department of Neurology, Hugo W. Moser Institute at Kennedy Krieger, 707 N. Broadway, Baltimore, MD 21205, USA; 2 Program in Human Genetics, Johns Hopkins School of Medicine, Baltimore, Maryland 21205, USA; 3 Program in Biochemistry, Cellular and Molecular Biology, Johns Hopkins School of Medicine, Baltimore, Maryland 21205, USA; 4 Department of Neuroscience, Johns Hopkins School of Medicine, Baltimore, MD 21205, USA; 5 Lilly Corporate Center, Eli Lilly and Company, Indianapolis, Indiana 46285, USA

## Abstract

**Background:**

Meiotic crossovers are the major mechanism by which haplotypes are shuffled to generate genetic diversity. Previously available methods for the genome-wide, high-resolution identification of meiotic crossover sites are limited by the laborious nature of the assay (as in sperm typing).

**Methods:**

Several methods have been introduced to identify crossovers using high density single nucleotide polymorphism (SNP) array technologies, although programs are not widely available to implement such analyses.

**Results:**

Here we present a two-generation "reverse pedigree analysis" method (analyzing the genotypes of two children relative to each parent) and a web-accessible tool to determine and visualize inheritance differences among siblings and crossover locations on each parental gamete. This approach is complementary to existing methods and uses informative markers which provide high resolution for locating meiotic crossover sites. We introduce a segmentation algorithm to identify crossover sites, and used a synthetic data set to determine that the segmentation algorithm specificity was 92% and sensitivity was 89%. The use of reverse pedigrees allows the inference of crossover locations on the X chromosome in a maternal gamete through analysis of two sons and their father. We further analyzed genotypes from eight multiplex autism families, observing a 1.462 maternal to paternal recombination ratio and no significant differences between affected and unaffected children. Meiotic recombination results from pediSNP can also be used to identify haplotypes that are shared by probands within a pedigree, as we demonstrated with a multiplex autism family.

**Conclusion:**

Using "reverse pedigrees" and defining unique sets of genotype markers within pedigree data, we introduce a method that identifies inherited allelic differences and meiotic crossovers. We implemented the method in the pediSNP software program, and we applied it to several data sets. This approach uses data from two generations to identify crossover sites, facilitating studies of recombination in disease. pediSNP is available online at .

## Background

Meiotic recombination or crossing over assures that each child inherits distinct genetic material from parental chromosomes. The process of meiotic recombination occurs between homologous chromosomes that pair and form chiasmata that promote normal chromosomal segregation during meiosis. In humans, mice, and other eukaryotes the sites of recombination characteristically occur in "hot spots" which are narrow regions of several thousand base pairs or less [1]. The Phase II HapMap annotates ~33,000 recombination hotspots that occupy approximately 6% of the genome sequence and account for ~60% of detectable crossovers in those individuals [2,3]. The number, distance between, and location of recombination sites vary considerably based on factors such as gender, age, and chromosome [4]. Meiotic crossovers can be considered in terms of chromosomal recombination occurring within a recent pedigree, or as haplotype blocks of high linkage disequilibrium (LD) that reflect the long-term recombination history of genomic regions.

Recombination rates have been determined using several main approaches: cytogenetics, direct molecular assays, and genetic linkage analysis [4]. Cytogenetics approaches allow chiasmata to be analyzed in meiocytes (spermatocytes or oocytes) in meiosis I. As an example of a direct assay, sperm typing has been used to determine genetic distances based on recombination, allowing the analysis of millions of sperm and high-resolution mapping of recombination hotspots [5]. Family-based linkage analysis has been used to determine recombination events in the parental generation [6]. Some approaches use three-generation pedigrees (e.g. [7]) to determine DNA segments that are shared identical by descent (IBD) between grandparent and grandchild, allowing identification of parental recombination events. Given informative polymorphic markers it is possible to identify meiotic crossover sites in two-generation pedigrees as was done, for example, in studies identifying recombination hotspots in the major histocompatibility complex [8,9] and the β-globin cluster [10].

While early studies employed up to hundreds of highly polymorphic markers, the introduction of SNP array technology allows from thousands to over a million markers to be assessed. Using an array with 11,454 SNP loci, Wirtenberger et al. (2005) implemented a schema (described below) to detect crossover regions in 16 members of a three-generation family. They separately compared the individual maternal and paternal haplotypes of a pair of siblings at all positions where the mother or the father were heterozygous. This allowed them to delineate regions of identical and nonidentical haplotype blocks that represented crossovers in the two siblings. More recently, Coop et al. [11] inferred recombination events in 725 related Hutterites. The Coop method involved analysis of pedigrees with both parents and two or more children, focusing on informative markers that allowed them to infer haplotypes transmitted from a parent to each offspring. For example, in some cases a heterozygous SNP in a father (i.e. an AB genotype call) and a homozygous SNP in a mother (e.g. an AA allele) can be informative (e.g. if the child is AB the B allele must be inherited paternally).

The determination of locations and patterns of meiotic recombination in pedigree data remains a challenge. We previously reported the SNPtrio program for the analysis of inheritance patterns in high density SNP genotype data [12]. SNPtrio analyzes genotype data from trios consisting of a father, mother and child. We now report a novel method to perform genome-wide mapping of meiotic recombination. This approach requires genotype data from pedigrees consisting of both parents and two or more children. The focus of the method is the use of "reverse trios" consisting of two siblings compared first to one parent and then to the other. We describe a web tool called pediSNP that processes genotype data from families with up to 10 children. These techniques enable the identification of shared or unique inheritance of chromosomal regions between siblings. Notably, this approach requires information from two-generation pedigrees (in contrast to some other approaches that rely on grandparental data for phasing), offers visualization of crossover regions, includes a segmentation method to identify blocks of shared alleles among siblings, and is capable of identifying maternally derived chromosome X crossovers when comparing two sons to their father. The father contributes no X material, so block structures are actually artifacts that reflect the maternal crossover events.

## Results

### Informative markers

**Figure 1:**
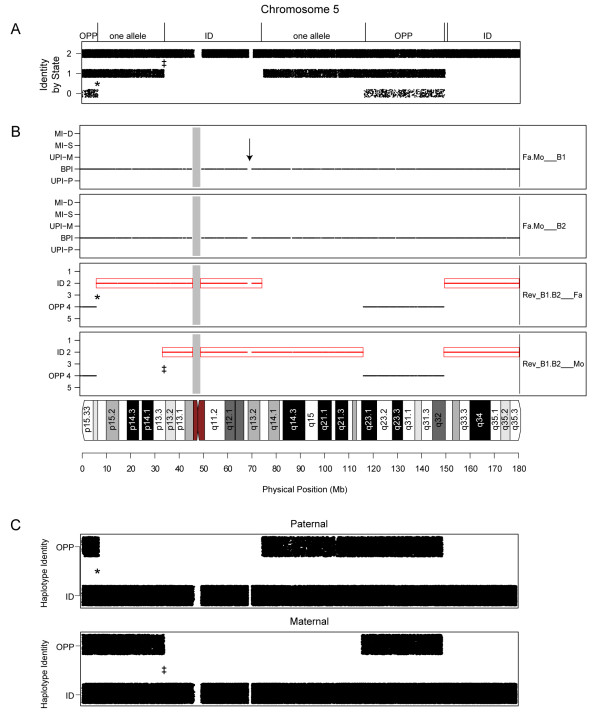
**Meiotic recombination in pedigrees with two children**. Analysis of meiotic recombination for chromosome 5 in two-generation pedigrees having two parents (Fa, Mo) and two children (B1, B2 for brothers 1 and 2). (A) Identity-by-state analysis in which each data point corresponded to a pairwise comparison between biallelic SNPs from siblings B1 and B2. This analysis showed regions of identical (ID) allele sharing, characterized by pairwise matches such as AA to AA for a given SNP. Regions in which the siblings had one shared allele included IBS1 signal (e.g. AA matching AB). Regions in which the siblings inherited opposite alleles included IBS0 in which there were zero shared alleles (e.g. AA aligned to BB). (B) pediSNP analysis. Panels 1 and 2 show SNPtrio results from normal trios (provided as part of the pediSNP analysis) of Fa/Mo/B1 and Fa/Mo/B2. The y-axis labels include Mendelian inconsistencies that are double (MI-D) or single (MI-S), uniparental inheritance that is maternal (UPI-M) or paternal (UPI-P), and biparental inheritance (BPI). Panels 3 and 4 show the results form "reverse pedigree" of B1/B2/Fa and B1/B2/Mo. By inspection of panels 3 and 4, distinct patterns are evident including identical inheritance (note two overlapping blocks from the ID 2 track), opposite inheritance (note overlapping blocks from the OPP 4 track), and one shared allele (note ID 2 pattern in one panel aligned with a blank region in the other panel). Rarely, a region is completely blank (arrow). This may be caused by the absence of SNP data (as at a centromere), the sharing of one allele by the parents (IBS 1), or the occurrence of "no calls" in that region due to homozygous deletion. (C) Implementation of the Wirtenberger et al. schema (defined in Additional File 4) showed patterns of allele sharing that were consistent with those identified by pediSNP. For all three approaches in panels A-C the edge of each block revealing allele sharing corresponded exactly to the nearest informative SNP (see positions marked *, ‡).

SNPtrio uses a schema to define inheritance patterns in genotype data from trios [12]; Additional File 1). There are five basic patterns detected by SNPtrio, plotted as tracks (see Figure 1B below) and also provided as tabular output. (1) Mendelian inconsistencies (MI) can consist of a double error (MI-D; e.g. father/mother/child genotypes of BB/BB/AA). (2) MI can also occur as a single error (MI-S; e.g. AA/AA/AB). Each occurrence of these aberrant patterns is plotted by SNPtrio on a track as a function of chromosomal position. MI SNPs occur only rarely and typically reflect genotyping errors or de novo genotype changes in the child. (3) Biparental inheritance (BPI) occurs in the common case in which the parents are homozygous with opposite alleles and each transmits one allele to the child (i.e. AA/BB/AB or BB/AA/AB for father/mother/child). For a typical high density SNP array approximately 5% of all autosomal SNPs within a trio comparison demonstrate biparental inheritance. (4) Paternal or (5) maternal uniparental inheritance (UPI-P or UPI-M) occurs when both of a child's alleles appear to originate from a single parent (e.g. AA/BB/AA, AA/BB/BB, or AA/AB/BB). SNPtrio provides evidence for uniparental hetero- or isodisomy (distinguished in the SNPtrio output as hUPI or iUPI denoting hetero- or iso-uniparental inheritance). Alternatively, a hemizygous deletion in a child results in an apparent uniparental inheritance pattern, and the inclusion of copy number data as part of the SNPtrio analysis facilitates the interpretation of such events as true UPD or genotype call artifacts due to copy number alteration.

SNPtrio was programmed to analyze genotype data from trios in the format father/mother/child. In the present work we applied an adaptation of the SNPtrio algorithm with "reverse pedigrees" in the format child1/child2/father and child1/child2/mother with the capacity to analyze pedigrees with at least two and as many as ten offspring. It should be noted that data are entered as standard pedigrees and the program automatically generates the appropriate reverse comparisons. We applied the SNPtrio schema to the reverse pedigrees, and assigned novel interpretations in order to identify regions in which the children have inherited the same (or different) parental haplotypes (Table 1). Of the categories of SNP patterns used by SNPtrio, two types are relevant to mapping recombination sites: BPI and MI-S. In reverse pedigrees these represent SNPs with opposite (OPP) and identical (ID) parental haplotypes in the two children, respectively (Additional Files 2 and 3). For example, the occurrence of patterns AA/AA/AB or BB/BB/AB for a child1/child2/parent1 reverse trio indicates that the two children inherited identical alleles (relative to each other) from that parent. (From the "forward trio" perspective of father/mother/child1 these genotypes would constitute MI-S.) For patterns AA/BB/AB or BB/AA/AB in child1/child2/parent1 reverse trios the children inherited opposite alleles from parent1.

**Table 1:** PediSNP plotting schema

**Child1**	**Child2**	**Parent**	**PediSNP track**
AA	AA	AA	None (noninformative)
AA	AA	AB	Track 2 (Identical)
AA	AA	BB	Track 1
AA	AB	AA	None (noninformative)
AA	AB	AB	None (noninformative)
AA	AB	BB	Track 3
AA	BB	AA	Track 5
AA	BB	AB	Track 4 (Opposite)
AA	BB	BB	Track 3

AB	AA	AA	None (noninformative)
AB	AA	AB	None (noninformative)
AB	AA	BB	Track 5
AB	AB	AA	None (noninformative)
AB	AB	AB	None (noninformative)
AB	AB	BB	None (noninformative)
AB	BB	AA	Track 5
AB	BB	AB	None (noninformative)
AB	BB	BB	None (noninformative)

BB	AA	AA	Track 3
BB	AA	AB	Track 4 (Opposite)
BB	AA	BB	Track 5
BB	AB	AA	Track 3
BB	AB	AB	None (noninformative)
BB	AB	BB	None (noninformative)
BB	BB	AA	Track 1
BB	BB	AB	Track 2 (Identical)
BB	BB	BB	None (noninformative)

The schema shows all 27 allele combinations for pedigrees with child1/child2/parent1. Two allele combinations signify identical inheritance by the children (AA/AA/AB and BB/BB/AB). The child1/child2/parent1 combinations AA/BB/AB and BB/AA/AB are informative in pediSNP, denoting opposite inheritance of alleles by the two siblings with respect to a parent. pediSNP implements the schema described in this table and in Additional File 1. In most circumstances the other SNP combinations occur only rarely (e.g. AA/BB/BB in child1/child2/parent1 reflects either a genotyping error or uniparental inheritance) or are not plotted (e.g. AA/AA/AA in child1/child2/parent1 is considered noninformative and is not plotted).

### Identification of inheritance differences in pedigrees with two children

The output of pediSNP for chromosome 5 of a pedigree consisting of both parents (Fa, Mo) and two children (brothers B1 and B2) contains four panels as well as a chromosomal ideogram (Figure 1B). The first two panels show the analysis results of standard SNPtrio for father/mother/B1 and father/mother/B2. Both SNPtrio panels contained a preponderance of black dots on the BPI tracks indicating that there were no large-scale deletions, duplications, or uniparental inheritance phenomena (such as uniparental disomy) in the children. The lower two panels show the results of reverse pedigree analysis for B1/B2/father and B1/B2/mother. Here the y-axis tracks are labeled 1-5 with ID (for identical inheritance) on track 2 (red dots) and OPP (for opposite inheritance) on track 4 (black dots). When examined together, these four panels can be used to infer regions of inheritance that occur in several prominent patterns: (1) identical inheritance (an IBD-2 region between siblings), (2) opposite haplotype inheritance (an IBD-0 region between siblings), (3) one shared allele (an IBD-1 region between siblings), and (4) autozygosity or deletion. We note that the pediSNP program requires three individuals to generate each panel of output, with individual data points plotted along tracks within the panel as a function of chromosomal position. At least two separate analyses are performed in parallel: child1/child2/father and child1/child2/mother. These analyses are independent from an algorithmic point of view, and the outputs are analyzed together to interpret the significance of which alleles are shared by the siblings.

(1) The two reverse pedigree plots display overlapping boxes with track 2 (ID) patterns indicated with red dots. This is a region where the parent contributed DNA sequences from the same physical chromosome to both siblings. Therefore, these two siblings have inherited DNA sequences that are identical by descent on that parental haplotype. The identical genotypes of each sibling are described by the pediSNP schema (Table 1) and by pedigrees (Additional File 2). The assignment of a region as identical was confirmed using identity-by-state (IBS) analysis (Figure 1A). Here we perform a pairwise comparison of SNP data from two siblings, and mark the track IBS-0 for each SNP having zero shared alleles (e.g. AA in one sibling and BB in the other), IBS-1 for one shared allele (e.g. AA/AB in sibling1/sibling2), or IBS-2 for two shared alleles (e.g. AA/AA in sibling1/sibling2). The region of identical inheritance shown by pediSNP corresponded to IBS-2 regions. This IBS analysis is part of the SNPduo program [13] and is not part of pediSNP but serves to illustrate its output.

(2) Both reverse pedigree plots contain a SNP pattern on track 4 (indicated with black OPP dots) denoting inheritance of non-identical alleles in the siblings for that parent. In this case, both parents contributed non-identical haplotypes to these two siblings. Thus, these two siblings did not share any single allele. This is referred as *opposite inheritance *region. The corresponding pedigree is shown in Additional File 3. In this case a comparison of the siblings includes blocks of IBS-0 (Figure 1A), indicating overlapping opposite haplotype inheritance regions.

(3) One parent's reverse pedigree plot is part of a red track 2 box while the other parent's plot has a blank space in the corresponding region. This pattern indicates that for the parent with a red track 2 box the two siblings inherited the same allele, and for the parent with blank space the two siblings inherited two separate alleles. This is a *semi-identical inheritance *region with only one parental haplotype shared identically by descent. The corresponding IBS plot included IBS-1 patterns as expected for one shared allele (Figure 1A).

We note that in Figure 1B, panel three, the pediSNP output for brother1/brother2/father includes a region on track 2 from about 8 Mb to 73 Mb, indicating that the brothers share an identical paternal allele. The analysis of brother1/brother2/mother includes a blank region with no data points plotted from the same starting position at 8 Mb and extending to 32 Mb. We infer that there is one shared allele from 8 to 32 Mb (as labeled at the top of Figure 1A). In this region, the brother1/brother2/father genotypes that are plotted as informative for track 2 are AA/AA/AB or BB/BB/AB. At each chromosomal position across this entire region, we note that there are no data points (red dots) in the brother1/brother2/mother panel, that is, the patterns AA/AA/AB or BB/BB/AB never occur. We observed empirically in this data set and in a large number of other data sets (data not shown) that these blank regions always lack genotype calls of the pattern child1/child2/father/mother AA/AA/AB/AB or BB/BB/AB/AB. The reason is that, in this example of child1/child2/mother never having observed AA/AA/AB, the mother cannot have a B allele since the children have no B alleles. The mother also cannot have a single A allele since that would necessitate that the children inherited an identical allele from the mother; this is impossible because the children have inherited an identical allele from the father, and if they were to also inherit an identical allele from the mother this would correspond to a region of two shared alleles, not one.

(4) All four panels (two usual trios and two reverse pedigree trios) can be blank, indicating no informative BPI or MI-S SNPs for SNPtrio (Figure 1B, arrow). There are several causes of these blank regions. One cause is autozygosity (relatedness by descent) in the parents. In such cases the parents share one or two alleles identical by descent in these regions. The shared alleles in the parents are treated as noninformative by the SNPtrio and pediSNP algorithms. Alternatively, there may be regions of the "no call" genotype in one or more individuals, due to homozygous deletion. Finally there may be a region where no SNPs are represented on the genotyping platform, such as a centromere or region of repetitive DNA. The arrow in this case represents a region without SNP coverage.

The schema employed by pediSNP is closely related to but distinct from those used by Wirtenberger et al. and Coop et al. (see Additional File 4). For pediSNP informative SNPs are identified based upon combinations of heterozygosity in one parent of a trio and homozygosity in two children, revealing opposite or identical inheritance of alleles; subsequent analyses using the other parent and the same two children reveal whether zero, one, or two alleles are shared. Of 27 possible child1/child2/parent1 combinations only four are informative, and thus pediSNP analyzes relatively few markers. For Coop et al. the approach requires heterozygosity in one parent and homozygosity in the other parent, with any zygosity in the child; subsequent analyses require data from the parents and an additional child. 12 father/mother/child genotype combinations are informative. The emphasis of the Coop et al. approach is parallel analyses of father/mother/child1 then father/mother/child2, in contrast to the pediSNP "reverse pedigrees" of child1/child2/father and child1/child2/mother. For Wirtenberger et al. the schema resembles the Coop approach, employing parallel analyses of father/mother/child with respect to maternal and paternal haplotypes. 14 father/mother/child combinations are informative (8 of these are in common with the Coop et al. schema; see Additional File 4). For example, father/mother/child AA/AA/AA is treated as noninformative by pediSNP and by Coop et al. but is used by Wirtenberger et al. to infer that and A allele must be transmitted from each parent to each child (Additional File 4). We implemented the Wirtenberger schema (Figure 1C) and showed that it described haplotype blocks in patterns that exactly matched those observed with both pediSNP (Figure 1B) and IBS analyses implemented with SNPduo [13] (Figure 1A). The schema employed by pediSNP is complementary to other approaches and yields comparable results in terms of crossover locations. The particular SNP combinations that are employed by pediSNP are useful to visualize a variety of genetic phenomena (such as maternal crossovers based on analyses of the male X; see below) and are intimately related to SNPtrio analyses that show uniparental inheritance phenomena (such as uniparental iso- or heterodisomy) or hemizygous deletion regions in SNP data [12].

Inheritance differences between two siblings can be mapped from these four types of blocks throughout the genome. However, given only two siblings we cannot determine which child inherited a given crossover.

### Identification of meiotic recombination sites in pedigrees with three or more children

**Figure 2:**
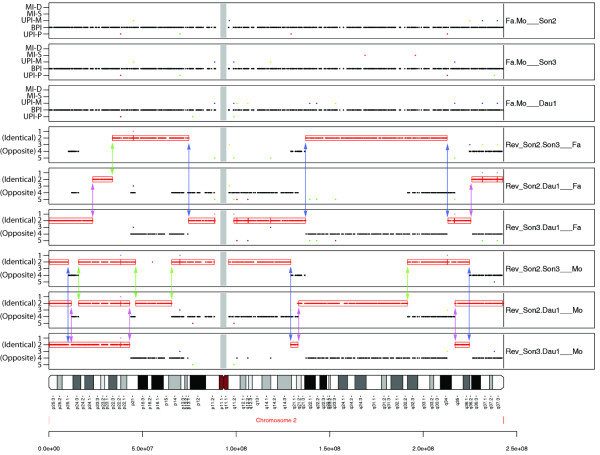
**Meiotic recombination in pedigrees with three children**. Identification of meiotic recombination events in two-generation pedigrees having two parents and three children. Seventeen meiotic recombination events were identified via pediSNP on chromosome 2 for three children of Autism Genetic Resource Exchange (AGRE) family AU1098. The top three panels correspond to conventional trios consisting of Father/Mother/Son2, Father/Mother/Son3 and Father/Mother/Daughter1. They contained the expected preponderance of BPI signals. The following six panels are "reverse" pedigrees for all three combinations of the two children versus father (panels 4-6) and versus the mother (panels 7-9). A vertical pink line with arrows identifies a meiotic recombination site of Daughter1, on a parent-specific allele, as indicated by the panel's label of father (middle three tracks) or mother (bottom three tracks). Lime colored lines denote crossovers in Son2 and blue colored lines denote Son3. The child who had a recombination event can be identified by the common individual (Child1 or Child2 of two separate Child/Child/Parent panels) of that event. For example, the Son2/Son3/Father trio ("Rev_Son2.Son3___Fa''; panel 4) and Son3/Daughter1/Father ("Rev_Son3.Dau1___Fa'' (panel 6) indicate that three separate crossover events occurred on Son3's paternal gamete (see three blue arrows in panels 4 and 6). In each of these three cases, an arrow points to the edge of a red track 2 box in two separate panels.

Using pediSNP, the analysis of pedigrees consisting of both parents and three children produced three standard SNPtrio panels (for the two parents relative to each of the three children) and six reversed pedigree panels (three for two children relative to the father and three for two children relative to the mother). An example is shown in Figure 2 for a pedigree from a multiplex family with autism including a daughter (Dau1) and two sons (Son2, Son3). The top three panels display the SNPtrio results of normal pedigrees, the next three panels show the results of reverse pedigrees with two siblings relative to the father, and the bottom three panels show reverse pedigree results relative to the mother.

Analysis of the three reverse pedigree panels associated with each parent allowed the determination of meiotic crossover sites in each child. The edges of identical inheritance regions (track 2 ID regions) aligned into pairs (with the exception of the telomeric and centromeric regions that lacked SNPs). We infer that each meiotic crossover caused a change in the block type pattern of that child compared to the other children for the parent in which the crossover occurred. For example, the first vertical pink line with arrows in Figure 2 (panels 5 and 6, left side) indicates an alignment between the trios of Son2/Daughter1/Father and Son3/Daughter1/Father at the edges of red track 2 ID boxes. Therefore, Daughter1 had a crossover on her paternal gamete in the region between the end of one block type and the beginning of another, indicated by the pink line. In principle the same alignment could have been caused by both Son2 and Son3 each inheriting a crossover in the same region (but not Daughter1). The likelihood of this scenario depends upon both on the marker density and the presence of hotspots in the region (see synthetic data section below). With four or more children these scenarios are more easily differentiated with high confidence. The output of pediSNP includes visualizations (such as those shown in Figure 2) and tabular summaries of the data that permit precise determination of the putative crossover regions. The physical position of each recombination event is located within the smallest gap between the red ID block and the closest black OPP block.

**Figure 3:**
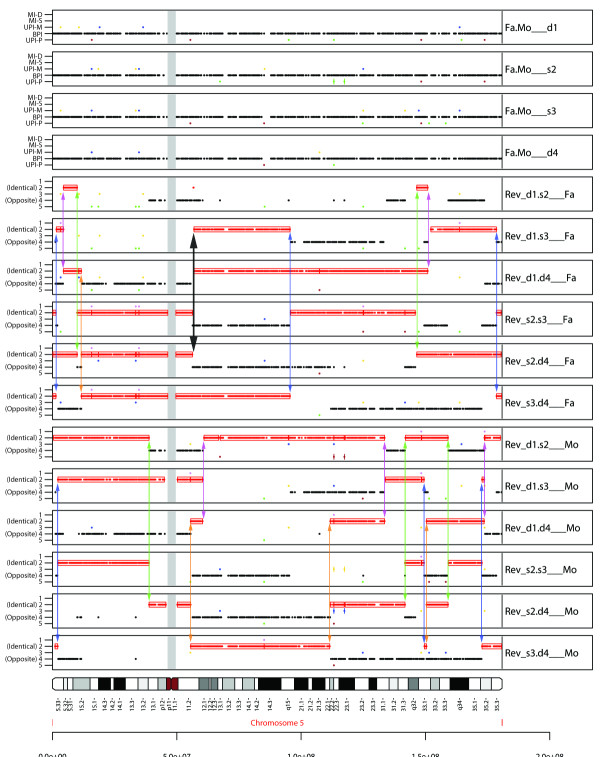
**Meiotic recombination in pedigrees with four children**. Meiotic recombination sites, on chromosome 5, identified for a family of parents with four children. The top four tracks are normal father-mother-child trios, for each of the four children (d1, s2, s3, d4). The following six tracks are reverse pedigrees for two children with respect to the father. The last six tracks are the reverse pedigrees for the mother's gamete. Vertical lines colored pink, green, blue and orange denote meiotic crossover sites of the child d1, s2, s3 and d4, in that order. The thick black line with arrows denotes a site where two of the four offspring had a meiotic recombination event on their paternal gamete with an additional crossover on the maternal gamete. In this case, the crossover events could have happened on either d1 and s2, or on s3 and d4.

With four children, pediSNP generates four standard SNPtrio panels and twelve reverse trio panels (six for each parent) as shown in Figure 3. For each parent, the ID regions in three reverse pedigree panels align to indicate a meiotic recombination event happened on that parent's gamete for the child who is in common to all those three trios. In some regions, two offspring will appear to have had meiotic recombination at the same site; we address this below. We note an especially complex situation in which there appear to be crossovers occurring within the same region of informative SNPs in both maternal and paternal gametes in multiple children (Figure 3, thick arrow).

### Segmentation and identification of recombination sites

The graphical output of pediSNP can be used to infer meiotic crossover sites. This can be done manually by visual inspection of the output to determine breakpoints in the tabular data (see Figures 1, 2 and 3 above). Crossover regions can be located considering one sibling with respect to his or her father or mother, and locating edges of blocks that occur at the same site in comparison to all other siblings. We implemented an algorithm to perform segmentation and automatically predict crossover sites (see Methods for details). Briefly, the algorithm first segments OPP and ID SNPs into blocks for each reverse comparison. For any given number of siblings, the algorithm then systematically analyzes each child relative to the parents to locate intervals between block edges that are shared with all other siblings. We observed that intervals overlap in spatially discrete clusters along a chromosome (occupying small distances) and encompassed the locations of known recombination sites in synthetic data. We compiled the genomic coordinates of all interval edges across each chromosome and identified the narrowest region of overlap (see Methods).

**Figure 4:**
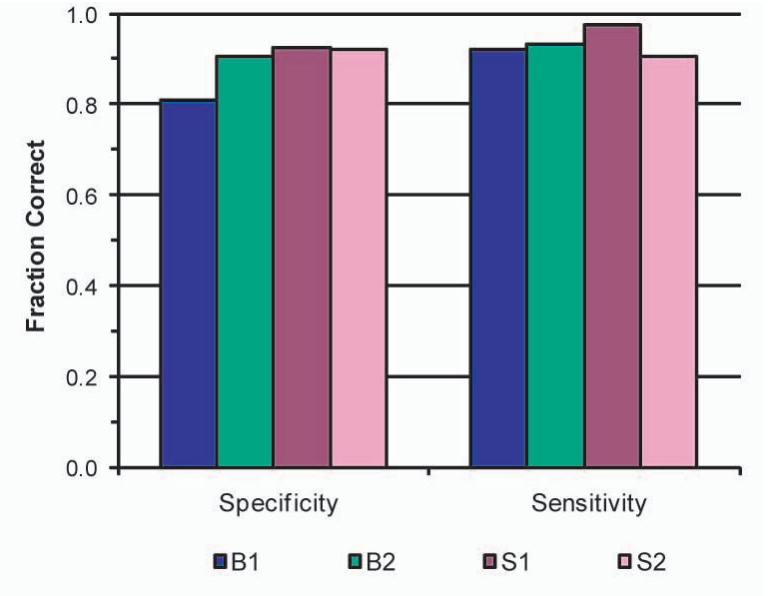
**Specificity and sensitivity of an algorithm for segmentation and identification of meiotic crossovers**. The performance of the segmentation and recombination identification algorithm was assessed using a synthetic data set consisting of three generations derived from HapMap genotype data. We determined the number of crossovers called by the algorithm compared to the number of actual recombinations in each individual. Plot shows representative results of one synthetic dataset (similar results were obtained with a second, independent synthetic data set). Data are shown for two brothers (B1, B2) and two sisters (S1, S2). Combining data from the four siblings, the mean ± standard deviation for specificity was 89.0% ± 0.6% and for sensitivity was 93.3% ± 0.3%.

We assessed the sensitivity and specificity of the recombination site prediction algorithm by using a pedigree synthetically derived from unrelated HapMap individuals (see Methods), including genotype data from three generations, and in which all crossover sites were known. A crossover site was defined as "found" if the region of minimum overlap included the known crossover location. The algorithm exhibited approximately 89% sensitivity and 92% specificity (Figure 4). Thus, 89% of known synthetic crossovers were correctly identified, and out of the sites the algorithm predicted to be recombination sites 92% were correct. We obtained similar results with a second synthetic pedigree (data not shown).

We investigated the causes underlying the false positive and false negative calls. Out of about 238 calls 214 were true synthetic recombination sites; 18 were false positive calls, and 24 true sites were not called (Table 2). Six of the predicted sites could not be phased to either the maternal or paternal gamete. The majority (55%) of false positive calls were caused by reflection of actual recombinations in one parental gamete in comparisons which included the other parent. The pediSNP approach of analyzing a pattern of SNPs inherited by two children relative to a parent reveals block-like structures, and when blocks are also evident in corresponding analyses of the other parent's gamete we refer to this as a reflection. Of these only 20% (11% of all false positives) were not properly called in the correct gamete. The remaining false positive calls (44%) were the result of situations in which large overlapping intervals caused erroneous calls to be made. Of the actual crossover sites that were missed (false negatives), 58% were missed because two synthetic recombinations occurred within a small genomic region in two synthetic children from one parent's gamete. This appears to be a limitation of using SNP data to identify recombination sites; implementation of a different schema for identifying inheritance patterns (Wirtenberger et al.) did not improve our ability resolve multiple crossover events that occurred in a very small region in separate children. Three out of the 24 missed calls (13%) were close to the beginning or end of a chromosome and fell within a region of ambiguity for the segmentation algorithm; four more were identified as recombination sites, but were of ambiguous parental origin, and the remaining three were not called because of large overlapping intervals confounding detection.

**Table 2:** False positive and false negative error rates

**Category**	**Type**	**Count**	**Percent**
False Positive Calls	Simple False Positives	8	44%
False Positive Calls	Reflected Duplication	8	44%
False Positive Calls	Reflected Overlap	2	11%
False Positive Calls	Total	18	100%
Missed Calls	Too Close	14	58%
Missed Calls	Overlapping Intervals	3	13%
Missed Calls	Ambiguous	4	17%
Missed Calls	Chromosome Ends	3	13%
Missed Calls	Total	24	100%

Analysis of false positive calls and false negatives (crossovers that were missed by the algorithm). Simple false positive refers to false positive calls that were not a result of comparisons to one parent reflecting events occurring in the other parent. Reflected Duplication false positive calls were predicted crossovers called in both parents at the same site, when there was only recombination in one parent. Reflected Overlap refers to erroneous calls being the result of intervals overlapping as a result of reflected events. Too Close refers to a recombination inherited by one sibling being very close to a second recombination inherited by a different sibling, but transmitted from the same parent. Overlapping Intervals that resulted in false positive calls often also resulted the inability to make the correct call. Ambiguous calls occurred when a call was made at the same region in both parents, thus the parent of origin cannot be phased; ambiguous calls were reported by the algorithm, but were not counted as having been detected. Chr Ends refers to actual recombination sites that were within ~250 Kb of the ends of the chromosomes; this algorithm cannot resolve crossovers that occurred in this region.

The segmentation algorithm does not include a genotype error model, but the occurrence of genotyping errors has only a small effect on the segmentation process (see Methods for details).

### Meiotic recombination in eight multiplex autism families

550 K Illumina SNP data for eight families were obtained from the Autism Genetic Resource Exchange (AGRE) and analyzed via pediSNP (summarized in Table 3 with detailed crossover results in Additional File 5). Each family had three to five children, with two or three children diagnosed with autism or autism spectrum disorder and least one other child without such diagnosis. We analyzed the genotypes of 28 children: 18 males (12 with autism, six non-autistic) and 10 females (four with autism, one with autism spectrum disorder, five non-autistic).

**Table 3:** Meiotic recombination sites in eight families

		**Average**		**Total**	**Mat:Pat**		**Mbp per recommendation**				**Autism**		**Unaffected**	
**chr**		**Pat**	**Mat**		**Ratio**		**Pat**	**Mat**	**Total**		**Pat**	**Mat**	**Pat**	**Mat**
chr1		1.54	3	4.54	1.95		161	82.42	54.51		1.53	2.82	1.55	3.27
chr2		1.75	2.89	4.64	1.65		138.83	83.98	52.33		1.71	2.59	1.82	3.36
chr3		1.86	2.71	4.57	1.46		107.42	73.5	43.64		1.76	2.59	2	2.91
chr4		1.29	2.25	3.54	1.75		148.77	85.01	54.1		1.24	2.35	1.36	2.09
chr5		1.64	2.43	4.07	1.48		110.09	74.47	44.42		1.65	2.41	1.64	2.45
chr6		1.32	2	3.32	1.51		129.33	85.45	51.45		1.18	1.88	1.55	2.18
chr7		1.43	2.18	3.61	1.53		111.17	72.9	44.03		1.53	2.24	1.27	2.09
chr8		1.21	2.21	3.43	1.82		120.46	66.06	42.66		1	2.12	1.55	2.36
chr9		1.25	1.61	2.86	1.29		112.22	87.28	49.1		1	1.41	1.64	1.91
chr10		1	2.14	3.14	2.14		135.37	63.17	43.07		1.12	2.12	0.82	2.18
chr11		1.43	1.61	3.04	1.13		94.12	83.66	44.29		1.41	1.71	1.45	1.45
chr12		1.25	1.79	3.04	1.43		105.88	74.12	43.6		1.47	2.12	0.91	1.27
chr13		0.93	1.43	2.36	1.54		122.92	79.9	48.42		0.94	1.41	0.91	1.45
chr14		0.96	1.04	2	1.07		110.31	102.7	53.18		1	1.18	0.91	0.82
chr15		1	1.43	2.43	1.43		100.34	70.24	41.32		1.06	1.65	0.91	1.09
chr16		1.07	1.39	2.46	1.3		82.91	63.77	36.05		1.06	1.18	1.09	1.73
chr17		1.21	1.36	2.57	1.12		64.87	58.04	30.63		1.18	1.18	1.27	1.64
chr18		1.07	1.39	2.46	1.3		71.04	54.65	30.89		1.06	1.35	1.09	1.45
chr19		0.75	0.93	1.68	1.24		85.08	68.72	38.02		0.59	0.82	1	1.09
chr20		1	1.11	2.11	1.11		62.44	56.39	29.63		1.06	1.18	0.91	1
chr21		0.5	0.82	1.32	1.64		93.89	57.15	35.53		0.53	0.59	0.45	1.18
chr22		0.82	0.75	1.57	0.91		60.49	66.26	31.62		0.94	0.82	0.64	0.64
per autosome		1.19	1.75	2.94							26	37.71	26.73	39.64
autosomes		26.29	38.46	64.75	1.46		109.1	74.56	44.29		26	37.71	26.73	39.64
chrX		0	1.64	1.64				94.3			0	1.29	0	2.18
chrY		0	0	0							0	0	0	0
all chr		26.29	40.11		1.53									

Summary of the number of meiotic recombination sites located with pediSNP in eight multiplex families with autism.

Abbreviations: chr, chromosome; Mat, maternal; Mbp, megabase pair; Pat, paternal.

Across all autosomes, the average numbers of meiotic recombination events identified on the paternal and maternal gametes of these twenty eight children were 26.29 and 38.43, respectively. The average Mbp per recombination for paternal and maternal gametes was 109.10 and 74.56, respectively. The average number of meiotic recombination events per autosome was 1.19 on the paternal gamete and 1.75 on the maternal gamete. The maternal to paternal ratio for autosomes was 1.462. With chromosome X included (see below), the genome-wide maternal to paternal recombination ratio was 1.526. These ratios of recombination events here (1.462/1.526 for autosomes and the genome, respectively), are comparable with previous studies: 1.555/1.625 for genetic length (cM) ratios in [6], 1.653/1.722 for genetic length (cM) ratios in [14]; 1.378/1.416 for crossover regions in [15]; and 1.511/1.721 for recombination events in [11].

The average number of autosomal recombination events among the seventeen affected children was 26.00 on the paternal gamete and 37.71 on the maternal gamete, similar to the averages from the eleven non-autistic children: 26.73 on the paternal gamete and 39.64 on the maternal gamete. The differences between affected and unaffected children were not significant on a combined genome-wide or chromosome-by-chromosome basis (p = 0.337), nor on the paternal gamete (p = 0.700) or maternal gamete (p = 0.479).

### Detection of maternal X chromosome recombination in sons

Recombination on the X chromosome occurs in females but not on the hemizygous male X. Recombination can occur between the pseudoautosomal regions PAR1 and PAR2 of the male X chromosome and the Y chromosome, although both PAR and Y chromosome SNPs are represented only sparsely on most commercial SNP platforms. The properties of female recombination are similar to those of other autosomes (e.g. [6,14,16-18]). We observed an average of 1.64 recombinations on the maternal X chromosome by measuring crossovers in reverse trios consisting of children compared to their mother (Table 3). We also inferred crossover events on the maternal gamete by analyzing reverse pedigrees consisting of two sons compared to their father (who has an X chromosome unrelated to that of his children). To our knowledge this is a unique feature of pediSNP.

**Figure 5:**
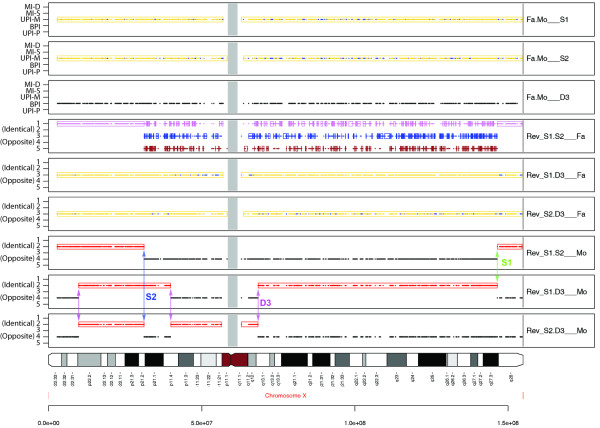
**pediSNP analysis of the X chromosome**. This pedigree included two parents (father Fa and mother Mo), two sons S1 and S2, and a daughter D3. The bottom three panels are marked with the five recombination events identified by pediSNP among three maternal gametes. The middle three panels do not contain any of the informative markers that occur on tracks 2 and 4, consistent with the lack of recombination events on any of those three paternal gametes. However, the fourth panel (Rev_S1.S2__Fa) which contains data from two brothers and the unrelated X chromosome of their father, included a set of patterns on tracks 1, 3, and 5 that reflect meiotic recombination events in the maternal gametes (see text for details). The top two panels show standard SNPtrio analyses, including a pattern of BPI for two parents relative to their daughter and exclusive maternal inheritance of the X chromosome by each of the sons.

An example is shown in Figure 5 for chromosome X of family AU1255 which has two sons and a daughter. The top three panels show standard SNPtrio results for the three children. For the parents compared to the daughter, the panel shows BPI signals exclusively, consistent with the inheritance of one copy of X from each parent. For each of the sons, the panels show signals in the maternal uniparental inheritance (UPI-M) track exclusively. (For the genotype patterns that generate the yellow and blue UPI-M signals see Additional File 1.) This is expected since the father does not contribute X chromosome material to the sons. The bottom three panels of Figure 5 show that for the maternal gamete analysis Son1 and Son2 each had one crossover event, while Daughter3 had three crossovers. The middle three panels of Figure 5 show the results of paternal gamete analysis. There were none of the typical blocks of informative SNPs for pediSNP (such as signals in tracks 2 and 4 in each panel) because the X chromosome of the father was unrelated to that of the sons. For the trios of Son1/Daughter3/Father or Son2/Daughter3/Father, the only signals that occurred were from track 3 (UPI-M in SNPtrio), reflecting the relationship of the daughter's X to her father's X. For the trio of Son1/Son2/Father (Figure 5, fourth panel), the output consisted of two types of regions: (1) signal from track 1 (MI-D in SNPtrio), and (2) signal from tracks 3 and 5 (UPI-M and UPI-P in SNPtrio). While the X chromosome of the father is unrelated to that of the two sons, the son's X chromosomes were identical to each other in regions of track 1 (MI-D) signal only. For example, the pattern in Son1/Son2/Father of AA/AA/BB or BB/BB/AA resulted in the track 1 (MI-D) signal and represented identical genotypes in the sons. In the region consisting of tracks 1, 3 and 5 signals, the brothers inherited different X chromosome haplotypes due to maternal recombination. This analysis was consistent with the results shown on the bottom three panels of Figure 5. The results were also consistent with the pairwise analysis of the genotypes based on IBS (Additional File 6). The identically shared telomeric regions consisted of IBS-2 exclusively, while the centromeric regions included additional IBS-0 signals that matched the track 1, 3 and 5 patterns shown on Figure 5 (fourth panel).

### Identification of candidate disease loci

With the ability to identify inheritance differences between any two siblings, applying pediSNP to families with both affected and unaffected children could reveal loci of interest, by locating regions of inherited alleles that segregate with the phenotype. Such an approach is inherently applied in linkage and association studies. The reverse pedigree approach can delineate genomic loci shared by probands that are more likely to harbor inherited mutations.

**Figure 6:**
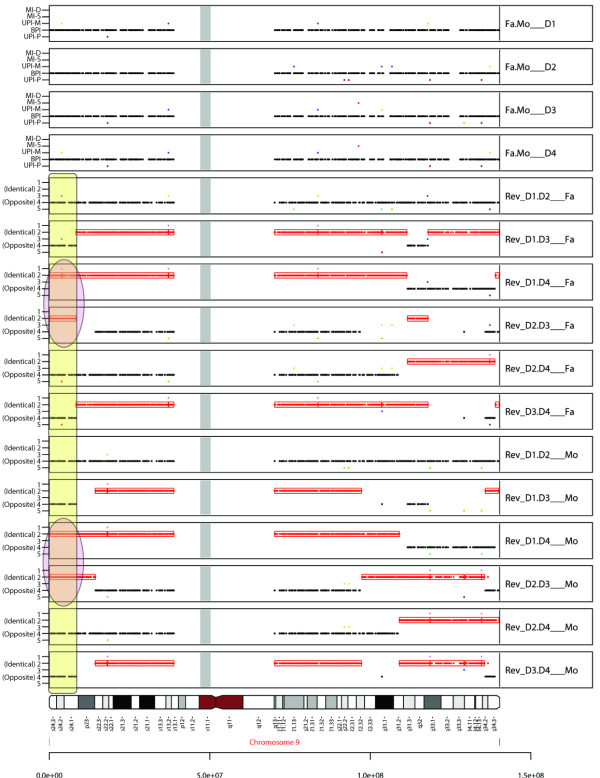
**Meiotic crossovers in multiplex autism families**. Analysis of chromosome 9 showing two probands with autism having shared alleles that are not inherited by unaffected siblings. We used pediSNP to analyze genotype data from a family with two parents (father Fa, mother Mo) and four daughters (probands D1 and D4, unaffected siblings D2 and D3). The top four panels (1-4) show the conventional SNPtrio output for the four father/mother/daughter trios. Panels 5-10 show the reverse trios of each pair of daughters relative to the father. The bottom six panels (11-16) show the reverse trios of daughters relative to the mother. The yellow box at 9p indicates a region where the two affected daughters had inherited identical alleles from both parents (red ovals) and those two alleles were the opposite alleles inherited by the unaffected daughters. Throughout the genome in multiplex families, such regions are relatively rare. For this families, only two such regions were found: 9p24 (shown in this figure), and part of 16p13.3.

As an example of the use of pediSNP to define loci of interest in a multiplex family with autism, we analyzed the AGRE pedigree AU1043. This family has four daughters: daughters 1 (D1) and 4 (D4) were diagnosed with autism and daughters 2 (D2) and 3 (D3) were diagnosed unaffected. pediSNP was used to generate a series of 16 panels of all chromosomes. Results are shown for chromosome 9 (Figure 6) and chromosome 16 (Additional File 7). Each plot contains four standard SNPtrio panels, six panels of reverse trios for the four offspring relative to the father, then six panels of reverse trios relative to the mother. We identified regions in which the two affected daughters (D1 and D4) shared alleles that were identical to each other, while the unaffected daughters (D2 and D3) inherited alleles identical to each other but opposite from the affected siblings. Thus the reverse trios contained red ID boxes (panels 7, 8 and 13, 14 of Figure 6 and Additional File 7). Furthermore, each of the remaining reverse pedigrees contained black OPP dots, indicating that the affected and unaffected offspring inherited opposite alleles. Genome-wide, inheritance differences between the affected and unaffected children identified by pediSNP included regions on 2p, 5q, 9p, 10q, 11q, 14q, 16p, 18p, 18q, 19q and 20q of the paternal gamete, and on 1q, 2p, 2q, 3q, 4q, 5q, 6q, 7p, 9p, 9q, 10p, 10q, 11p, 11q, 12q, 14q, 15q, 16p, 17p and 22q on the maternal gamete. (The loci on 2p, 5q, 10q, 11q and 14q corresponded to two different regions on the parents.) The regions on 9p and 16p were identical between the two parents: 9p24.1-24.3 (Figure 6) and 16p13.3 (Additional File 7). The region of 9p24.3 has recently been identified as one of five regions of interest in an extended pedigree of seven autism probands and 22 relatives [19]. 16p13.3 was also identified as a region implicated for autism because the GABA-transaminase gene is located there [20].

## Discussion

It is important to understand the nature of human recombination for many reasons including its role in generating genetic diversity and the effect of aberrant recombination in causing aneuploidy, a leading cause of both miscarriages and disorders such as Down syndrome. Recombination history generates patterns of linkage disequilibrium, which is the correlation or covariance between neighboring SNPs.

The reverse trio approach that we introduce in this paper uses a schema to define informative SNPs that originated with the SNPtrio tool [12]. Using "reverse pedigrees" of two children relative to a parent, we were able to detect identical, opposite, and semi-identical haplotypes. The pediSNP schema uses a relatively small set of informative markers (Table 1). This contrasts with the schemas described by Coop et al. and Wirtenberger et al. (Additional File 4) that have been used to infer meiotic crossovers and have employed markedly different sets of informative markers and a higher density of markers. Nonetheless the pediSNP schema performs similarly in its identification of crossovers (see e.g. Figure 1), and it offers several benefits. (1) Because the "reverse pedigree" analysis is performed in the context of the standard SNPtrio analysis, one can investigate the relationship between chromosomal abnormalities (such as hemizygous or homozygous deletions or UPD) and meiotic crossover sites. (2) It is web-based and thus accessible to those studying pedigrees. The web interface is accessible across operating systems and browsers. Other programs such as Merlin [21] assess crossovers, although not with visualization or reverse pedigree analysis. In common with PLINK [22], pediSNP can assess inheritance differences (loci of interest) between siblings. As an example of the utility of this approach, it is possible to examine a pedigree in which a child has inherited a recessive disease-associated allele and to use pediSNP to determine whether other siblings share this haplotype. (3) It relies on two generation pedigrees (in common with Wirtenberger et al. and Coop et al.). This offers an advantage relative to the reliance on three-generation pedigrees (see e.g. [6,7]) because from a practical point of view grandparental SNP genotypes are often difficult to obtain.

A novel feature of pediSNP is that it permits the analysis of crossovers on the maternal X chromosome, even when analyzing SNP patterns from one or more sons and the father (who has an unrelated X). Our approach allowed us to determine the maternal crossovers based on separate analyses of paternal and maternal genotypes. Notably for SNP-based methods, hemizygous SNPs (A or B), such as SNPs on a male X chromosome, are interpreted as homozygous (AA or BB) with most current genotype calling algorithms. This reduces the number of heterozygous calls and thus limits the ability to detect crossovers on the X chromosomes in males with alternative algorithms. For example, Coop et al. [11] provide data on the 22 autosomes but not the X chromosome.

## Conclusion

Meiotic recombination is an essential feature of chromosomal biology and is largely responsible for generating haplotype diversity in offspring. Until recently, methods to detect meiotic crossovers were either laborious (e.g. sperm typing) or required three generation pedigrees. We introduce a novel reverse pedigree algorithm to analyze crossover locations within pedigrees, and implement this method with the pediSNP program. This provides a user-friendly, accessible way to identify meiotic crossovers. We anticipate that this tool will be useful for studies related to disease (e.g. in identify haplotypes harboring mutations that are shared by siblings) or chromosomal biology (e.g. characterizing events such as crossover interference or variation in crossover frequency and location).

## Methods

The pediSNP program is publicly available [23] and was based on SNPtrio [12] (available at the same website). The pediSNP website includes the software, a tutorial, and text files containing all the genotype data used in this study.

SNP data based on Illumina's 550 K SNP chip were obtained from AGRE as Illumina BeadStudio data files. SNP data of genotype calls were exported from BeadStudio as text files, and used as the input to pediSNP. Forward trio analysis was performed by steps described in SNPtrio and plotted as trio tracks. Meiotic recombination sites were identified among reverse trio tracks of identical or opposite inheritance, based on patterns and descriptions shown in Figure 1.

Figure 1B shows typical output from the pediSNP analysis. The top two panels (labeled as 'Fa.Mo__B1' and 'Fa.Mo__B2') are from normal pedigree runs: the two parents with the sons B1 and B2, respectively. The next two panels (labeled as 'Rev_B1.B2__Fa' and 'Rev_B1.B2__D3') are from reverse pedigree analysis, with the two siblings treated as if they are the parents, and each of the two biological parents treated as if he or she is the child, respectively.

A unique dimension introduced by pediSNP lies in blocks of dots shown in both the OPP and the ID tracks (refer to Table 1) on the two reverse pedigree plots. Each black OPP (opposite inheritance) dot signifies that the parent of this reversed child1/child2/parent trio has the AB genotype while the children of this reversed trio include an AA genotype and a BB genotype. The child with the AA genotype inherited a copy of the A from that parent, while the child with the BB genotype inherited a copy of the B from that parent. A block of black OPP dots identifies a region where two non-identical alleles from that parent were transmitted separately to the two siblings.

Because any black OPP dot in one parent's reverse trio plot indicates that parent has an AB genotype while the two siblings have one AA genotype and one BB genotype, this means that the other parent must also have an AB genotype. Therefore, the dot on the other parent's reversed trio plot must also be a black OPP dot. (An exception is the occurrence of a chromosomal anomaly such as uniparental inheritance.) Thus, for example, in Figure 1 the two reverse trios have OPP blocks that are aligned in the region labeled "opposite inheritance."

Each contiguous red ID box region on any one of those two reversed trio plots identifies a segment of that particular chromosome where one of that parent's allele was not transmitted to either of the two siblings. For that chromosomal region, the two siblings inherited the same allele from this parent. The beginning and the end points of each such chromosomal region mark two different meiotic crossover events on that parent's chromosome among these two siblings. Each crossover was transmitted to only one sibling and not to the other sibling. Both crossover events could have been transmitted to the same child.

Note that OPP dots signify that parent1 is AB while one child is AA and the other child is BB. Consider a region of semi-identical (IBS-1) inheritance (Figure 1). When one of the parent's reverse pedigree plot has a red ID box region (i.e., these two siblings inherited the same allele from that parent), there will be no OPP dots on the corresponding space of the reverse pedigree plot from the other parental gamete, due to the sharing of an allele among the "parents" of this reverse pedigree. The matching pair of reverse pedigree plots could have overlapping red ID boxes, marking a region where each sibling inherited the opposite allele from each parent.

The SNPtrio tool can show paternal or maternal uniparental inheritance phenomena (UPI-P or UPI-M) such as hemizygous deletions, as well as Mendelian inconsistencies. SNPtrio panels are plotted as part of the pediSNP output. Similar patterns are occasionally evident on reverse trio plots. (For example, Figure 2 panels 2 and 3 each include one dot on the UPI-M track on the short arm of chromosome 2 adjacent to the centromere, and these dots are reflected on the tracks labeled 5 of panels 4 and 5.) In reverse trios such patterns are not informative regarding meiotic recombination.

### Segmentation algorithm

We developed a segmentation algorithm to define blocks and their overlaps. We first defined blocks having either opposite or identical patterns with a start and end position for the first and last informative SNP. We then ordered the blocks by starting position and defined overlapping blocks. For example, block 1 with ends A, B and block 2 with ends C, D overlap if A ≤ D and C ≤ B. Analyses of block overlaps were restricted to one parental gamete at a time, and were used to find n-1 overlapping blocks where n is the number of siblings. After overlapping blocks are identified, the algorithm identifies the maximum genomic distance within which the crossover event occurred. Crossovers are flagged as ambiguous if they are found to occur in two parental gametes. For overlaps in fewer than n-1 blocks, the criteria for calling a crossover are not met. All crossover calls are tabulated into a final output.

The segmentation algorithm does not include a genotype error model. Genotyping errors are not more likely to occur in parents or children, and Mendelian inconsistencies in father/mother/child trios are analyzed in the SNPtrio panels of pediSNP. The segmentation algorithm relies on data in tracks 2 (identical inheritance) and 4 (opposite inheritance) and is thus unaffected by genotyping errors which would generate signals in tracks 1, 3, or 5. The presence of a single signal in tracks 2 or 4 that was due to genotyping error would not disrupt the segmentation algorithm because it relies on locating the beginning and end of blocks to find the crossovers. A single MI-S or BPD in the middle of another block type would not generate enough points to create a block (and would be too far away from other blocks) to be considered. Furthermore, if there were by chance two or more dots interrupting a block, it could correspond to a true recombination event. This can be assessed by finding consensus among n-1 siblings. Even if it were found, an erroneous block would be ignored unless it matched in multiple individuals. For these reasons the segmentation algorithm is insensitive to single outliers (genotyping errors) and would only to start to be affected when there were multiple points clustered together. And then the clusters would only cause a problem if they were replicated between multiple siblings (e.g. the occurrence of parental errors of specific types and not sibling errors).

### Synthetic data generation

Synthetic SNP data were generated to test the segmentation algorithm's sensitivity and specificity using data in which recombination sites and gamete choices were known. Data were based on a HapMap genotype scaffold (n = 1,066,825 markers) and distributed throughout the genome at their annotated coordinates. A three generation pedigree was designed to include maternal and paternal grandparents, two parents, and four children/grandchildren.

The first step in creating the synthetic pedigree was to create the grandparents' genotypes from which all individuals would be derived. The synthetic parents for each grandparent were defined as two unrelated HapMap Yoruba individuals for which SNP data were available. The father/mother for the Paternal Grandfather, Paternal Grandmother, Maternal Grandfather, and Maternal Grandmother were individuals NA18507/NA18858, NA18871/NA18517, NA19138/NA19172, and NA19160/NA19116. The HapMap founders' genotypes were initially randomly segregated into haplotypes. Location and number of crossovers were determined for each individual. Each chromosome arm could have one or two crossover events, and the probability of each scenario was dependent on chromosome arm size and sex (Table 4), chosen to reflect a greater rate of crossing over in females.

**Table 4:** Crossover probabilities

**Arm size**	**Gender**	**Long arm**	**Short arm**
> 80 Mb	male	0.55	0.55
> 80 Mb	female	0.4	0.6
60-80 Mb	male	0.5	0.5
60-80 Mb	female	0.45	0.55
< 60 Mb	male	0.98	0.22
< 60 Mb	female	0.972	0.028

Sex-specific crossover probabilities based on the size of chromosome arms. Females were given a higher recombination rate than males, and larger arms had a greater probability of two crossovers than shorter arms did.

Once the number of recombinations was determined, crossover locations were determined. For each crossover a 1 Mb bin was selected based on the weighted sex-specific recombination probability [14] available from the UCSC genome browser [24]. Once a 1 Mb bin was selected the exact location within the bin was selected at random. If two crossovers were chosen a 10 Mb interference region was generated on either side of the first crossover region. The second crossover was selected using the same method as the first from the remaining pool of bins.

With crossover locations decided, the haplotypes were swapped in the appropriate positions to create a choice of two non-recombinant and two recombinant gametes for each chromosome. The choice of which gamete to transmit to progeny was independent for all chromosomes in females. Males did not recombine their X, and therefore could only transmit the X to female offspring or the Y chromosome male offspring. The probability of selecting a non-recombinant chromosome was 35%, while the probability of selecting a recombinant chromosome was 65%.

Once the synthetic gametes were generated for each founder pair, the gamete haplotypes were stored for the individual they created, and that individual's genotypes were derived from the inherited haplotypes. This ensured that the individuals in the first generation (the grandparents) had allele frequencies representative of the sampled population and had genotypes that were based on real data.

With the grandparents established, synthetic children were derived in a very similar manner, the only difference being that haplotypes were already established. Each crossover location and gamete choice was recorded in the output, giving a complete list of all of the crossovers that occurred to create each individual. The reported data were given as tabular genotypes annotated by chromosome and position for each member of the pedigree.

## Availability and requirements

• **Project name: **pediSNP

• **Project home page: **

• **Operating system(s): **Platform independent web interface, Linux only webserver

• **Programming language: **Perl, R, C, html

• **Other requirements: **none

• **License: **GNU GPL

• **Any restrictions to use by non-academics: **none

## List of Abbreviations

BPI: biparental inheritance; MI-D: double Mendelian inconsistency; MI-S: single Mendelian inconsistency; SNP: single nucleotide polymorphism; UPI: uniparental inheritance, OPP: opposite inheritance; ID: identical inheritance; IBS: identity-by-state; IBD: identity-by-descent.

## Competing interests

The authors declare that they have no competing interests.

## Authors' contributions

JCT developed the project, implemented the original software, and co-wrote the manuscript. EDOR optimized software, generated synthetic data, contributed to the segmentation algorithm development, and contributed to the writing of the manuscript. DGC programmed the segmenting algorithm, calculated sensitivity and specificity, and contributed to the writing of the manuscript. JP conceived the project and contributed to data analysis and writing the manuscript. All authors read and approved the final manuscript.

## Pre-publication history

The pre-publication history for this paper can be accessed here:



## Supplementary Material

Additional file 1 **Schema for SNPtrio**. The SNPtrio schema as reported in Ting et al. (2007). This schema includes AA, AB, or BB genotype calls for a trio consisting of two parents and one child. Informative SNP combinations are plotted on tracks (see e.g. Figure 1B). Abbreviations: MI-D, double Mendelian inconsistency; MI-S, single Mendelian inconsistency; BPI, biparental inheritance; iUPI-P, paternal uniparental isodisomy; hUPI-P, paternal uniparental heterodisomy; iUPI-M, maternal uniparental isodisomy; hUPI-M, maternal uniparental heterodisomy. The bottom of the table shows how the pediSNP schema is employed with trios consisting of two children and one parent, and in parallel analyses, the same two children and a different parent. The SNPtrio schema is applied to pediSNP with y-axis tracks 1-5 plotted as shown in Figure 1B. Track 2 corresponds to identical (ID) inheritance of alleles, while track 4 corresponds to inheritance of opposite (OPP) alleles. Click here for file

Additional file 2 **Single Mendelian inconsistencies (MI-S) and identical inheritance patterns in forward and reverse pedigrees**. (A) In forward trios, MI-S patterns occur when a child has a single allele not present in either parent (i.e. father/mother/child AA/AA/AB or BB/BB/AB). In this figure, as in Additional File 3, the three individuals analyzed are indicated with gray symbols. MI-S in forward trios tends to occur only rarely and is due to genotyping errors or mutations. (B) In reverse trios, the pattern AA/AA/AB or BB/BB/AB in child1/child2/mother is scored as track 2 (identical) according to the pediSNP schema. No MI has occurred, but rather the interpretation is that child1 and child2 have an identical, homozygous genotype (both AA or both BB) while the parent is heterozygous (AB). The SNPtrio program (Ting et al., 2007) performs a statistical test to determine when a genomic region contains a string of MI-S calls, generates a red box surrounding that region, and calculates a probability value for the likelihood of that string occurring by chance. In pediSNP, the red track 2 signals are surrounded by a red box that is interpreted as the two children sharing identical alleles. The track 2 signals observed in the reverse pedigree including the mother (as shown in the pedigree in this figure) are further interpreted in the context of signals observed in the reverse pedigree including the father in order to determine whether the siblings share identical or semi-identical alleles. Click here for file

Additional file 3 **Biparental inheritance (BPI) and opposite inheritance patterns in forward and reverse pedigrees**. (A) In forward trios, BPI occurs when the parents are opposite homozygotes, making the child an obligate heterozygote (i.e. in father/mother/child the genotypes of a given biallelic SNP are AA/BB/AB or BB/AA/AB; each trio used in the analysis consists of the three individuals indicated with gray symbols). BPI typically occur ~5% of the time in analysis of trios, and they are expected to occur across the genome unless there are interruptions by anomalies such as deletions or duplications. BPI signals are indicated as black dots. (B) In reverse pedigrees, genotypes of two children are analyzed relative to one parent (in this figure the gray symbols in each pedigree indicate the three individuals analyzed by pediSNP). The child1/child2/mother trios are shown both in the conventional pedigree format (with children at the bottom) or as reverse pedigrees with children at the top; these two representations are equivalent, offering two perspectives on the pediSNP schema. The output on track 2 ("opposite") in pediSNP includes black dots that only occur when the two children have homozygous SNPs with no shared alleles (i.e. AA/BB or BB/AA in child1/child2). Click here for file

Additional file 4 **Wirtenberger and Coop schemas**. Schemas for defining informative SNP patterns by Wirtenberger et al. and Coop et al. In the Wirtenberger schema, father/mother/child combinations are defined from which transmission from a maternal haplotype (column Wirt. hapM) or paternal haplotype (Wirt. hapF) are considered in two separate analyses at each SNP position. For example, for pattern AA/AA/AA it is inferred that the child inherited an A from each parent. The Wirtenberger schema includes additional analysis for missing data (not shown). For the Coop et al. schema, informative positions occur where one parent has a homozygous call and the other parent is heterozygous (e.g. AA/AB/AA)(see arrowheads). Click here for file

Additional file 5 **Meiotic crossover sites in eight autism families**. Meiotic crossover sites were located with pediSNP in eight autism families as a function of chromosome. Children identified with a single asterisk were diagnosed as having autism, while a double asterisk indicates a diagnosis of Autism Spectrum Disorder. Values represent the number of meiotic crossover events. In some instances it is possible to determine that a crossover has occurred, but it is not possible to unambiguously assign the crossover to a child or his/her sibling. In such cases we recorded a value of 0.5 crossovers for each sibling. Click here for file

Additional file 6 **Identity by state on the X chromosome**. IBS analysis of the X chromosome in two brothers (S1 and S2). While the male chromosome X is hemizygous, current SNP platforms interpret the genotype (A or B) as the biallelic calls AA or BB, and there are essentially no heterozygous (AB) calls. In the telomeric regions, these two males have an IBS 2 signal that corresponds to identically shared segments (i.e. AA matches AA or BB matches BB). In the central portion of the chromosome (physical postion ~31 Mb to ~147 Mb), an IBS 0 signal is present consistent with these two X chromosomes being unrelated across the region. The lower panels show the genotype calls. The results of this SNPduo analysis are consistent with those of pediSNP. Click here for file

Additional file 7 **Recombination in pedigrees with autistic probands**. Identification of shared alleles on chromosome 16p13.3 in probands with autism that are not inherited by unaffected siblings. The same analysis described in Figure 5 was applied to all chromosomes. The results for chromosome 16 are shown, including a region (green-shaded rectangle) that is shared identically by the two probands (red-shaded ovals) with inheritance of opposite alleles from the unaffected sisters. Click here for file
