# Supplementary material for: Locations and patterns of meiotic recombination in two-generation pedigrees
Source: BMC Med Genet. 2009 Sep 17;10:93. doi: 10.1186/1471-2350-10-93 (PMC2760526; doi:10.1186/1471-2350-10-93)
Supplement: Additional file 1 — Schema for SNPtrio. The SNPtrio schema as reported in Ting et al. (2007). This schema includes AA, AB, or BB genotype calls for a trio consisting of two parents and one child. Informative SNP combinations are plotted on tracks (see e.g. Figure 1B). Abbreviations: MI-D, double Mendelian inconsistency; MI-S, single Mendelian inconsistency; BPI, biparental inheritance; iUPI-P, paternal uniparental isodisomy; hUPI-P, paternal uniparental heterodisomy; iUPI-M, maternal uniparental isodisomy; hUPI-M, maternal uniparental heterodisomy. The bottom of the table shows how the pediSNP schema is employed with trios consisting of two children and one parent, and in parallel analyses, the same two children and a different parent. The SNPtrio schema is applied to pediSNP with y-axis tracks 1-5 plotted as shown in Figure 1B. Track 2 corresponds to identical (ID) inheritance of alleles, while track 4 corresponds to inheritance of opposite (OPP) alleles. [file 1471-2350-10-93-S1.DOC]

| **SNPtrio schema** | | | | | | | | | |
| --- | --- | --- | --- | --- | --- | --- | --- | --- | --- |
| Father | Mother | Child | iUPI-P | hUPI-P | BPI | hUPI-M | iUPI-M | MI-S | MI-D |
| AA | AA | AA |  |  |  |  |  |  |  |
|  |  | AB |  |  |  |  |  | x |  |
|  |  | BB |  |  |  |  |  |  | x |
|  | AB | AA |  |  |  |  |  |  |  |
|  |  | AB |  |  |  |  |  |  |  |
|  |  | BB |  |  |  |  | x |  |  |
|  | BB | AA | x | x |  |  |  |  |  |
|  |  | AB |  |  | x |  |  |  |  |
|  |  | BB |  |  |  | x | x |  |  |
| AB | AA | AA |  |  |  |  |  |  |  |
|  |  | AB |  |  |  |  |  |  |  |
|  |  | BB | x |  |  |  |  |  |  |
|  | AB | AA |  |  |  |  |  |  |  |
|  |  | AB |  |  |  |  |  |  |  |
|  |  | BB |  |  |  |  |  |  |  |
|  | BB | AA | x |  |  |  |  |  |  |
|  |  | AB |  |  |  |  |  |  |  |
|  |  | BB |  |  |  |  |  |  |  |
| BB | AA | AA |  |  |  | x | x |  |  |
|  |  | AB |  |  | x |  |  |  |  |
|  |  | BB | x | x |  |  |  |  |  |
|  | AB | AA |  |  |  |  | x |  |  |
|  |  | AB |  |  |  |  |  |  |  |
|  |  | BB |  |  |  |  |  |  |  |
|  | BB | AA |  |  |  |  |  |  | x |
|  |  | AB |  |  |  |  |  | x |  |
|  |  | BB |  |  |  |  |  |  |  |
| Child1 | Child2 | Parent1 |  |  | opp. inhert. |  |  | identical inherit. |  |
| y-axis track label on pediSNP graphical output | | | 5 | 5 | OPP 4 | 3 | 3 | ID 2 | 1 |
| **pediSNP schema** | | | | | | | | | |

Ting et al.

Additional File 1
