# Supplementary material for: Locations and patterns of meiotic recombination in two-generation pedigrees
Source: BMC Med Genet. 2009 Sep 17;10:93. doi: 10.1186/1471-2350-10-93 (PMC2760526; doi:10.1186/1471-2350-10-93)
Supplement: Additional file 2 — Single Mendelian inconsistencies (MI-S) and identical inheritance patterns in forward and reverse pedigrees. (A) In forward trios, MI-S patterns occur when a child has a single allele not present in either parent (i.e. father/mother/child AA/AA/AB or BB/BB/AB). In this figure, as in Additional File 3, the three individuals analyzed are indicated with gray symbols. MI-S in forward trios tends to occur only rarely and is due to genotyping errors or mutations. (B) In reverse trios, the pattern AA/AA/AB or BB/BB/AB in child1/child2/mother is scored as track 2 (identical) according to the pediSNP schema. No MI has occurred, but rather the interpretation is that child1 and child2 have an identical, homozygous genotype (both AA or both BB) while the parent is heterozygous (AB). The SNPtrio program (Ting et al., 2007) performs a statistical test to determine when a genomic region contains a string of MI-S calls, generates a red box surrounding that region, and calculates a probability value for the likelihood of that string occurring by chance. In pediSNP, the red track 2 signals are surrounded by a red box that is interpreted as the two children sharing identical alleles. The track 2 signals observed in the reverse pedigree including the mother (as shown in the pedigree in this figure) are further interpreted in the context of signals observed in the reverse pedigree including the father in order to determine whether the siblings share identical or semi-identical alleles. [file 1471-2350-10-93-S2.PPT]

## Slide 1
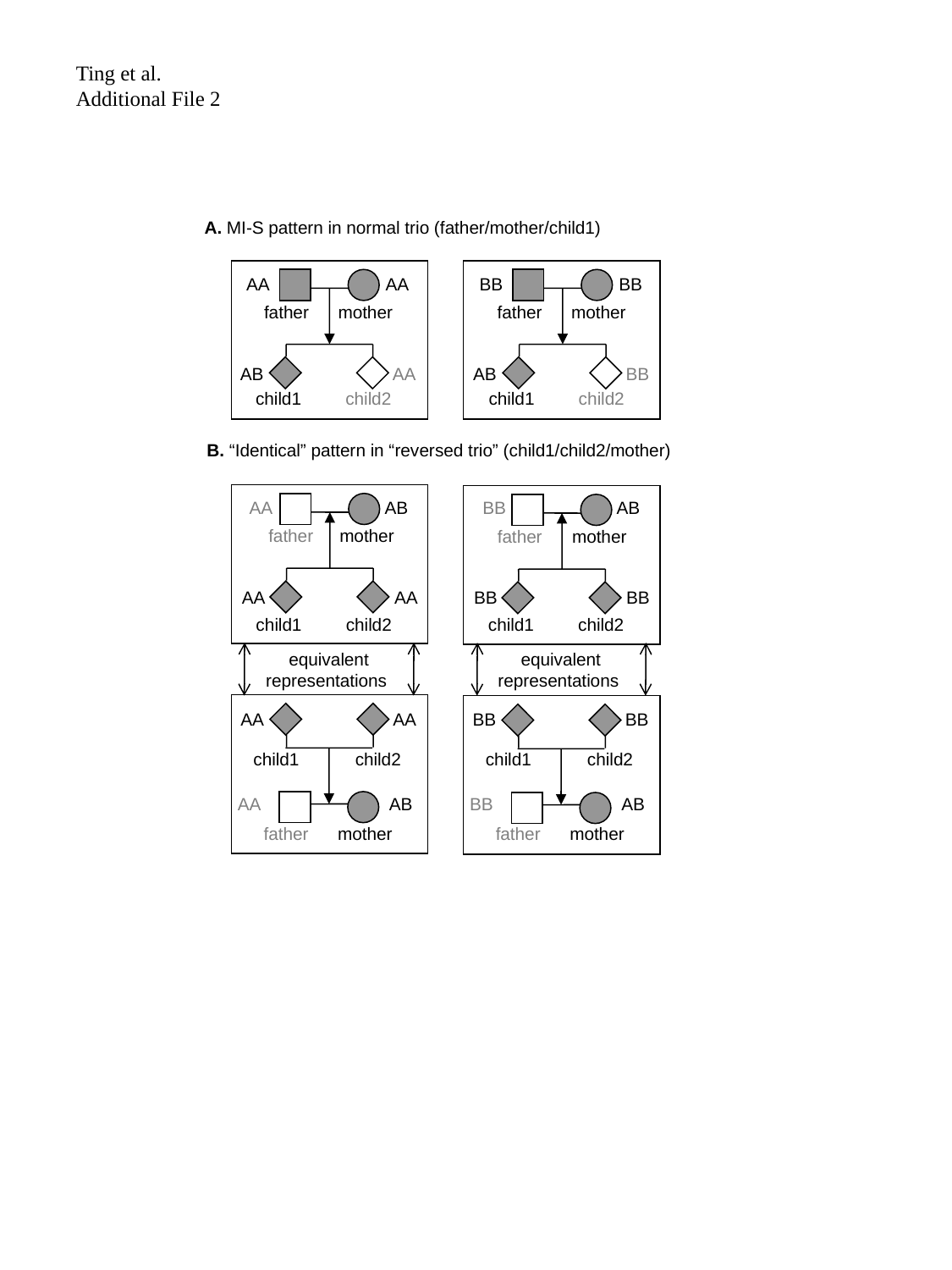

Ting et al.
Additional File 2
A. MI-S pattern in normal trio (father/mother/child1)
AA
AA
BB
BB
father
mother
father
mother
AB
AA
AB
BB
child1
child2
child1
child2
B. “Identical” pattern in “reversed trio” (child1/child2/mother)
AA
AB
BB
AB
father
mother
father
mother
AA
AA
BB
BB
child1
child2
child1
child2
equivalent
representations
equivalent
representations
AA
AA
BB
BB
child1
child2
child1
child2
AA
AB
BB
AB
father
mother
father
mother
