# Supplementary material for: Locations and patterns of meiotic recombination in two-generation pedigrees
Source: BMC Med Genet. 2009 Sep 17;10:93. doi: 10.1186/1471-2350-10-93 (PMC2760526; doi:10.1186/1471-2350-10-93)
Supplement: Additional file 3 — Biparental inheritance (BPI) and opposite inheritance patterns in forward and reverse pedigrees. (A) In forward trios, BPI occurs when the parents are opposite homozygotes, making the child an obligate heterozygote (i.e. in father/mother/child the genotypes of a given biallelic SNP are AA/BB/AB or BB/AA/AB; each trio used in the analysis consists of the three individuals indicated with gray symbols). BPI typically occur ~5% of the time in analysis of trios, and they are expected to occur across the genome unless there are interruptions by anomalies such as deletions or duplications. BPI signals are indicated as black dots. (B) In reverse pedigrees, genotypes of two children are analyzed relative to one parent (in this figure the gray symbols in each pedigree indicate the three individuals analyzed by pediSNP). The child1/child2/mother trios are shown both in the conventional pedigree format (with children at the bottom) or as reverse pedigrees with children at the top; these two representations are equivalent, offering two perspectives on the pediSNP schema. The output on track 2 ("opposite") in pediSNP includes black dots that only occur when the two children have homozygous SNPs with no shared alleles (i.e. AA/BB or BB/AA in child1/child2). [file 1471-2350-10-93-S3.PPT]

## Slide 1
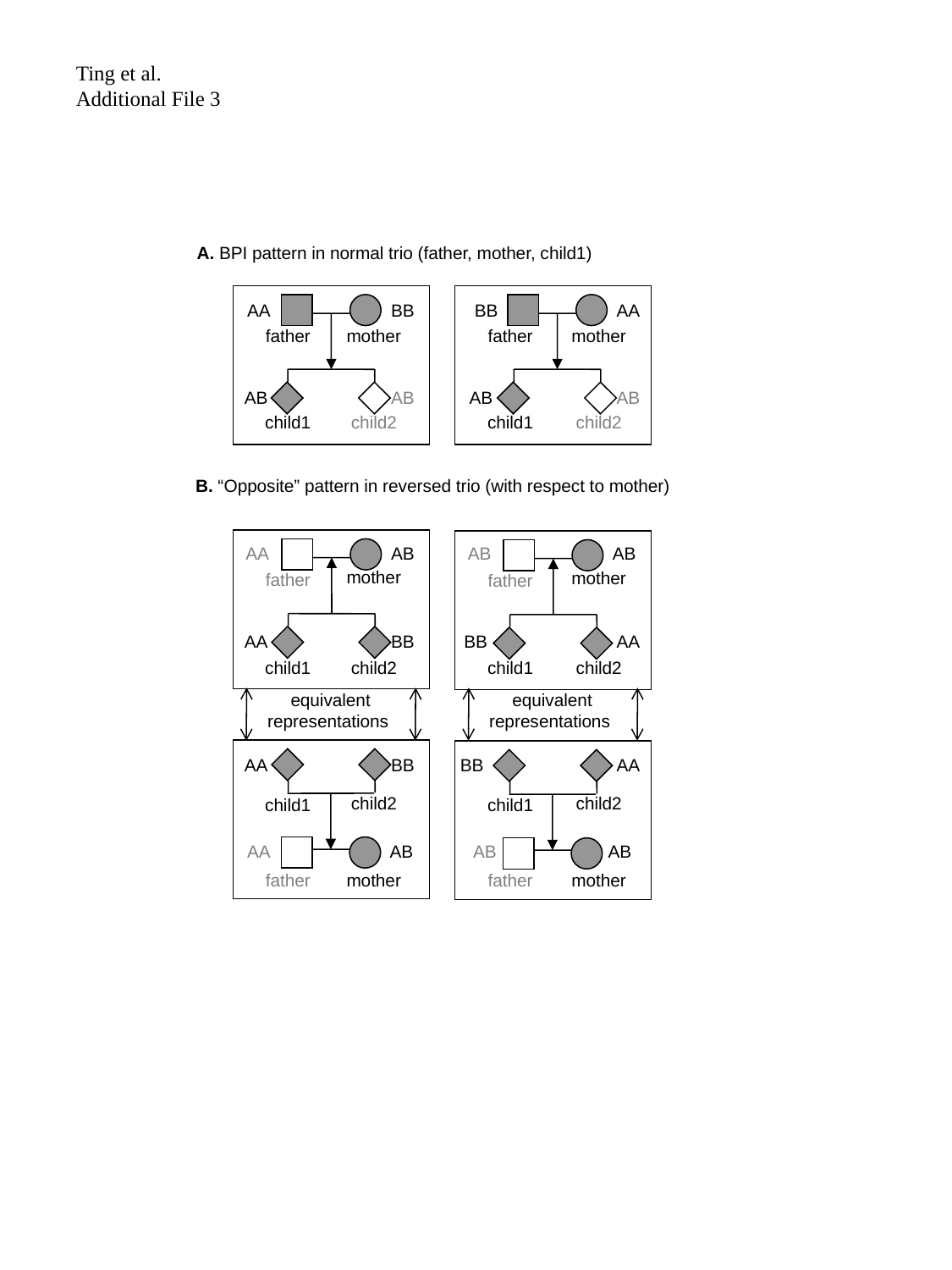

Ting et al.
Additional File 3
A. BPI pattern in normal trio (father, mother, child1)
AA
BB
BB
AA
father
mother
father
mother
AB
AB
AB
AB
child1
child2
child1
child2
B. “Opposite” pattern in reversed trio (with respect to mother)
AA
AB
AB
AB
mother
mother
father
father
AA
BB
BB
AA
child1
child2
child1
child2
equivalent
representations
equivalent
representations
AA
BB
BB
AA
child2
child2
child1
child1
AA
AB
AB
AB
father
mother
father
mother
