# Supplementary material for: Locations and patterns of meiotic recombination in two-generation pedigrees
Source: BMC Med Genet. 2009 Sep 17;10:93. doi: 10.1186/1471-2350-10-93 (PMC2760526; doi:10.1186/1471-2350-10-93)
Supplement: Additional file 4 — Wirtenberger and Coop schemas. Schemas for defining informative SNP patterns by Wirtenberger et al. and Coop et al. In the Wirtenberger schema, father/mother/child combinations are defined from which transmission from a maternal haplotype (column Wirt. hapM) or paternal haplotype (Wirt. hapF) are considered in two separate analyses at each SNP position. For example, for pattern AA/AA/AA it is inferred that the child inherited an A from each parent. The Wirtenberger schema includes additional analysis for missing data (not shown). For the Coop et al. schema, informative positions occur where one parent has a homozygous call and the other parent is heterozygous (e.g. AA/AB/AA)(see arrowheads). [file 1471-2350-10-93-S4.DOC]

Ting et al.

Additional File 4

| Father | Mother | Child | Wirt. hapM | Wirt. hapF | Coop |
| --- | --- | --- | --- | --- | --- |
| AA | AA | AA | A | A |  |
|  |  | AB |  |  |  |
|  |  | BB |  |  |  |
|  | AB | AA | A | A | ◄ |
|  |  | AB | B | A | ◄ |
|  |  | BB |  |  | ◄ |
|  | BB | AA |  |  |  |
|  |  | AB | B | A |  |
|  |  | BB |  |  |  |
| AB | AA | AA | A | A | ◄ |
|  |  | AB | A | B | ◄ |
|  |  | BB |  |  | ◄ |
|  | AB | AA | A | A |  |
|  |  | AB |  |  |  |
|  |  | BB | B | B |  |
|  | BB | AA |  |  | ◄ |
|  |  | AB | B | A | ◄ |
|  |  | BB | B | B | ◄ |
| BB | AA | AA |  |  |  |
|  |  | AB | A | B |  |
|  |  | BB |  |  |  |
|  | AB | AA |  |  | ◄ |
|  |  | AB | A | B | ◄ |
|  |  | BB | B | B | ◄ |
|  | BB | AA |  |  |  |
|  |  | AB |  |  |  |
|  |  | BB | B | B |  |
