# Supplementary figures and images for: Locations and patterns of meiotic recombination in two-generation pedigrees
Source: BMC Med Genet. 2009 Sep 17;10:93. doi: 10.1186/1471-2350-10-93 (PMC2760526; doi:10.1186/1471-2350-10-93)

## Slide 1
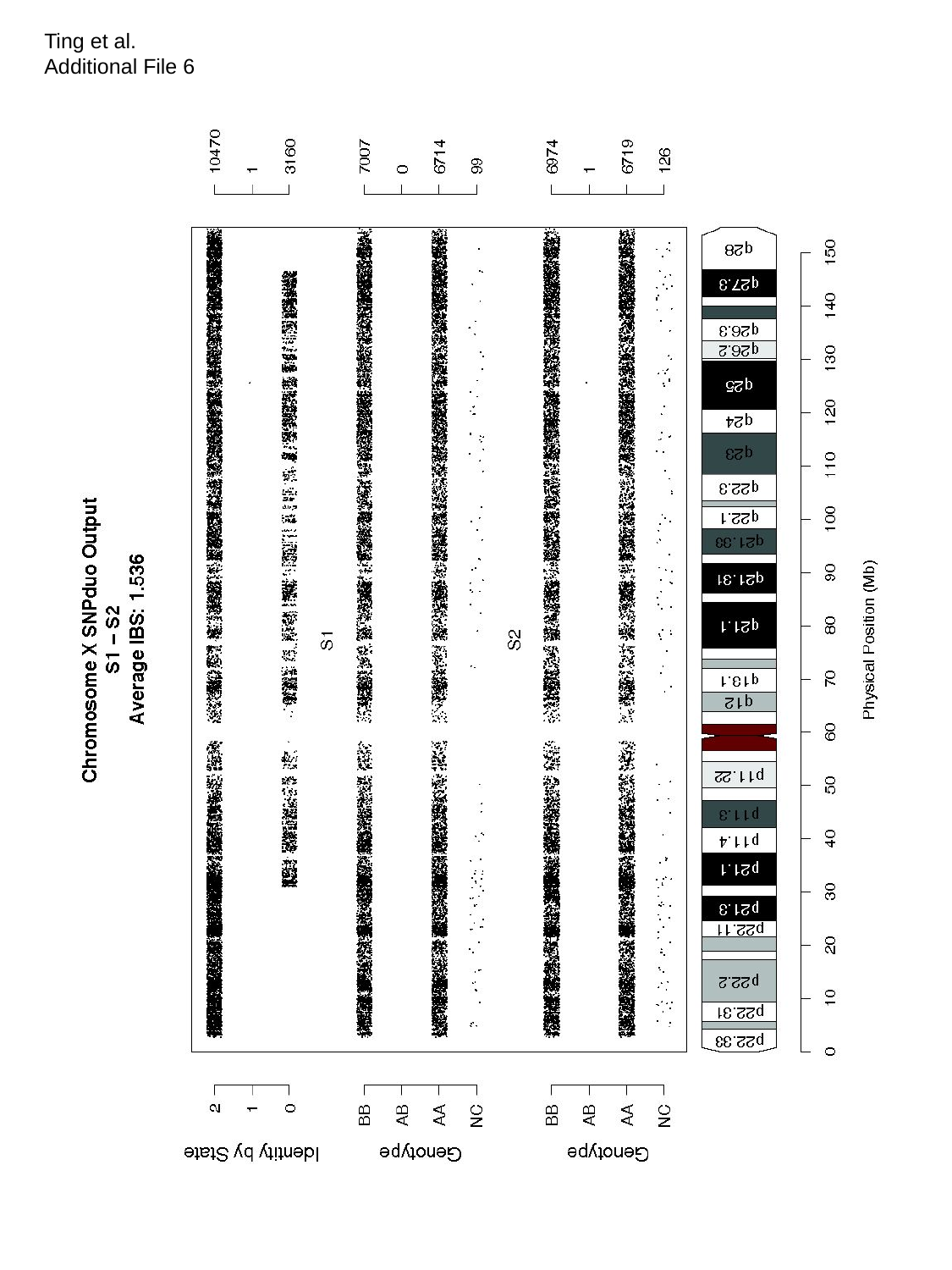

Ting et al.
Additional File 6

Supplement: Additional file 6 — Identity by state on the X chromosome. IBS analysis of the X chromosome in two brothers (S1 and S2). While the male chromosome X is hemizygous, current SNP platforms interpret the genotype (A or B) as the biallelic calls AA or BB, and there are essentially no heterozygous (AB) calls. In the telomeric regions, these two males have an IBS 2 signal that corresponds to identically shared segments (i.e. AA matches AA or BB matches BB). In the central portion of the chromosome (physical postion ~31 Mb to ~147 Mb), an IBS 0 signal is present consistent with these two X chromosomes being unrelated across the region. The lower panels show the genotype calls. The results of this SNPduo analysis are consistent with those of pediSNP. [file 1471-2350-10-93-S6.PPT]

## Slide 1
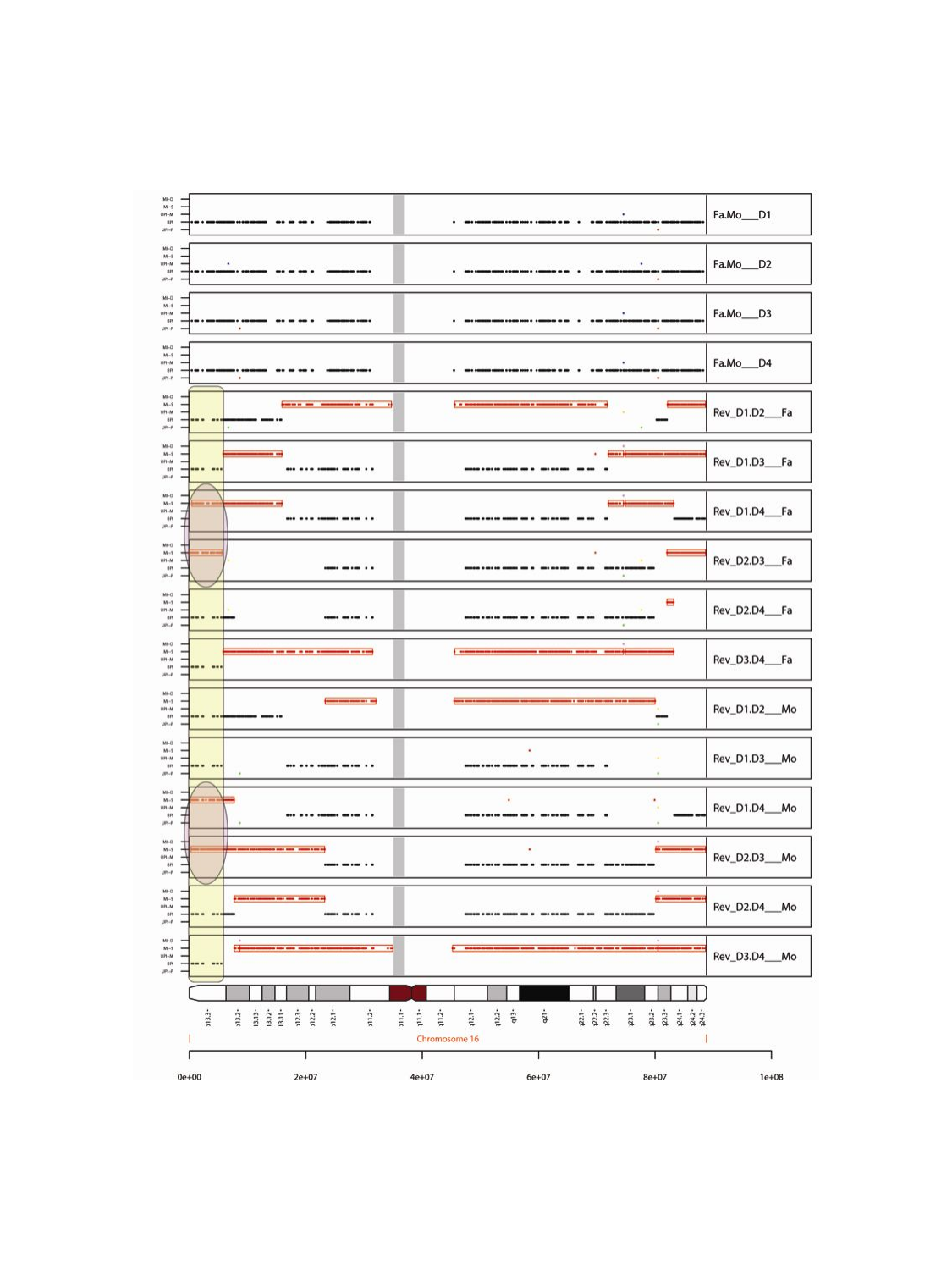

Supplement: Additional file 7 — Recombination in pedigrees with autistic probands. Identification of shared alleles on chromosome 16p13.3 in probands with autism that are not inherited by unaffected siblings. The same analysis described in Figure 5 was applied to all chromosomes. The results for chromosome 16 are shown, including a region (green-shaded rectangle) that is shared identically by the two probands (red-shaded ovals) with inheritance of opposite alleles from the unaffected sisters. [file 1471-2350-10-93-S7.PPT]
